# Into the breach: how cells cope with wounds

**DOI:** 10.1098/rsob.180135

**Published:** 2018-10-03

**Authors:** Mitsutoshi Nakamura, Andrew N. M. Dominguez, Jacob R. Decker, Alexander J. Hull, Jeffrey M. Verboon, Susan M. Parkhurst

**Affiliations:** Basic Sciences Division, Fred Hutchinson Cancer Research Center, Seattle, WA 98109, USA

**Keywords:** single cell wound repair, plasma membrane repair, calcium, membrane trafficking, actomyosin, Rho GTPases

## Abstract

Repair of wounds to individual cells is crucial for organisms to survive daily physiological or environmental stresses, as well as pathogen assaults, which disrupt the plasma membrane. Sensing wounds, resealing membranes, closing wounds and remodelling plasma membrane/cortical cytoskeleton are four major steps that are essential to return cells to their pre-wounded states. This process relies on dynamic changes of the membrane/cytoskeleton that are indispensable for carrying out the repairs within tens of minutes. Studies from different cell wound repair models over the last two decades have revealed that the molecular mechanisms of single cell wound repair are very diverse and dependent on wound type, size, and/or species. Interestingly, different repair models have been shown to use similar proteins to achieve the same end result, albeit sometimes by distinctive mechanisms. Recent studies using cutting edge microscopy and molecular techniques are shedding new light on the molecular mechanisms during cellular wound repair. Here, we describe what is currently known about the mechanisms underlying this repair process. In addition, we discuss how the study of cellular wound repair—a powerful and inducible model—can contribute to our understanding of other fundamental biological processes such as cytokinesis, cell migration, cancer metastasis and human diseases.

## 1. Introduction

**Figure 1.**
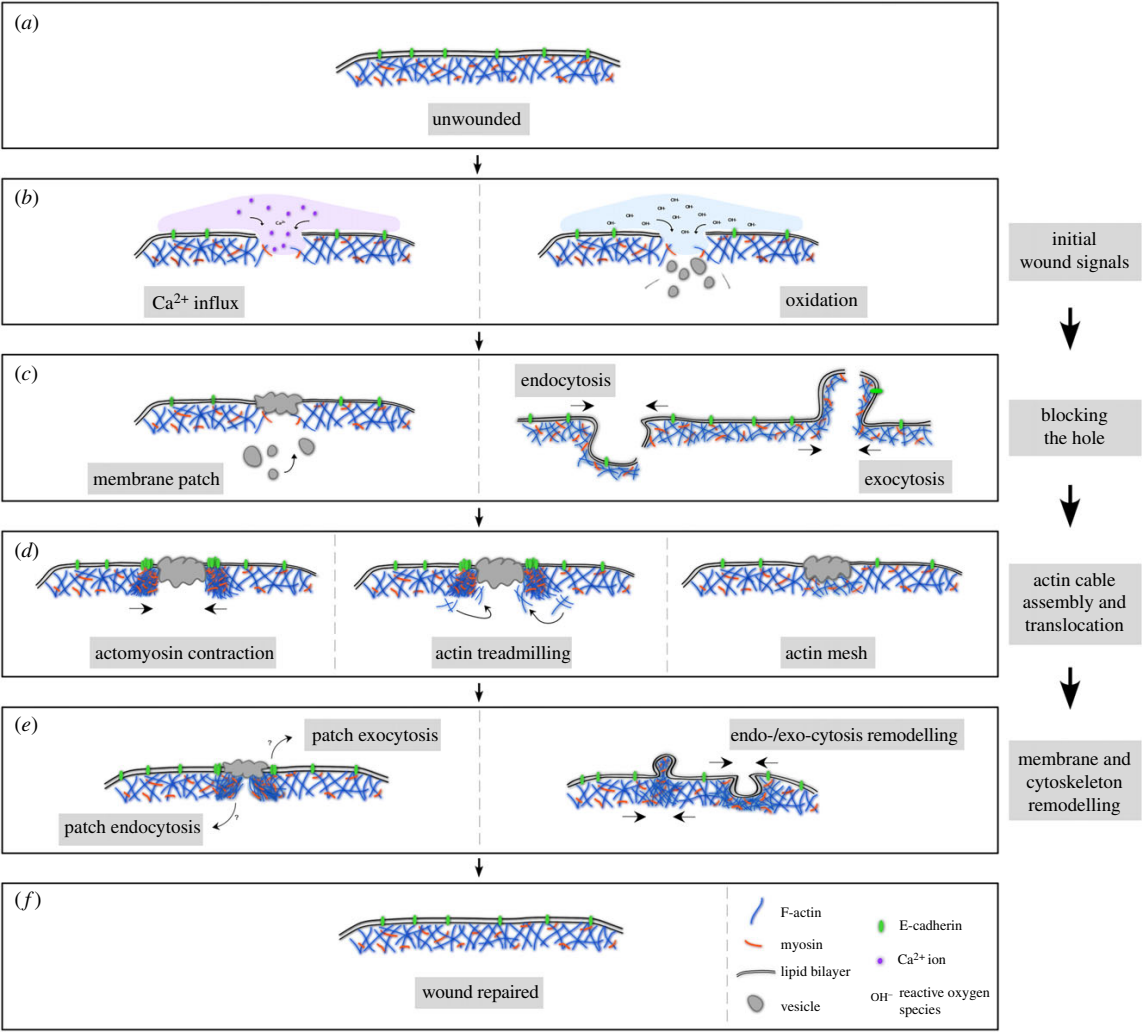
Schematic of major steps in cell wound repair. (*a*) Cross section of unwounded cell showing intact plasma membrane and underlying cortical cytoskeleton. (*b*) Cells with a membrane lesion showing influx of calcium ions (left panel) or reactive oxygen species (right panel) that oxidize cellular components. Influx of these ions each start signalling cascades that initiate cellular wound repair processes. (*c*) Cells quickly close wounds, either through a vesicular membrane patch (left panel), or by endocytosis/exocytosis of the area of membrane containing the lesion (right panel). (*d*) Actin based cytoskeleton works to close cellular wounds by an actomyosin contractile ring (left panel), actin treadmilling where the leading edge closest to the wound is the site of F-actin assembly and the trailing edge distal to the wound has F-actin disassembly (middle panel), or by a transient actin patch that accumulates under the membrane lesion (right panel). (*e*) Cells with repaired wounds must remodel their plasma membrane and cortical cytoskeleton to their pre-wounded state. The fate of the transient membrane patch is not well studied, but it is thought to be removed from the membrane by either endocytosis or exocytosis (left panel). In addition, components of the actin cytoskeleton, which are enriched at the wound site are thought to be either endocytosed or exocytosed in order to return that region to its pre-wounded state (right panel). (*f*) Cell with completely healed wound—plasma membrane and underlying cortical cytoskeleton have been returned to their unwounded state.

Cells have been observed to repair major disruptions to their plasma membranes for over a century, and the capacity for self-repair is fundamental to an organism's robustness [1–7]. Cells, like organisms, regularly need to repair wounds from a variety of acute stresses, as well as from day to day activity, and this complex response must match the scale of the damage inflicted and the specific type of wounding event. Decades of work on the subject in a variety of organisms and systems have revealed a generally conserved framework for wound closure, as well as highlighting how different mechanisms have been co-opted by different organisms to achieve the same end: wound repair (figure 1*a–f*). Despite much progress, major gaps in our understanding of this phenomenon remain.

Upon wounding, it is essential that the cell prevents the loss of interior components and cytoplasm, and prevents the entry of unwanted material, in order to maintain viability [1–5]. As such, it is critical that the cell is able to quickly detect a membrane breach and rapidly reseal the plasma membrane. Interestingly, despite the importance of minimizing the entry of extracellular components, much is still unknown about how cells sense they are wounded and initiate the repair process. In a variety of model systems and human/mammalian tissue culture cells, influx of extracellular calcium has been the earliest detected and presumed starting signal for cell wound repair (figure 1*b*) [5,8–12]. Recently, however, alternative wound repair starting signals have been proposed [13,14], suggesting that the means of wounding and/or the type/size of wounds may contribute to how cells initiate the repair process (figure 1*b*). This initiation of wound repair leads to rapid membrane resealing. Intriguingly, it appears that cells from different organisms may accomplish membrane resealing through different mechanisms [2,3,15]. One long held hypothesis is that vesicles gather and fuse at the site of the membrane lesion to form a transient membrane ‘patch’ (figure 1*c*) [11,16,17]. However, mounting evidence suggests that this may not occur in all cases as some cells appear to use mechanisms involving endocytosis/exocytosis (figure 1*c*) [8–10,15,18,19], and more recently it has been shown in *Xenopus* oocytes that a population of vesicles at the site of the wound are violently exocytosed, and resultant membrane fusions reseal the membrane hole [20].

Following this immediate ‘triage’, cellular wounds are repaired by constriction of the membrane and underlying cortical cytoskeleton followed by remodelling of the cell cortex, which returns the wounded site to its pre-wounded state (figure 1*d*) [2,21–23]. Extensive work in *Drosophila* embryos and *Xenopus* oocytes has demonstrated that actin-based dynamics, downstream of the classic Rho family GTPase cytoskeleton regulators, are critical to this aspect of wound repair. In the case of *Xenopus* oocytes, a ring of actin constricts around the wound by way of actin treadmilling (figure 1*d*) [24,25], whereas a contractile actomyosin ring promotes closure in *Drosophila* embryos (figure 1*d*) [22,26,27]. Interestingly, it has been shown in mammalian tissue culture cells that the actin cytoskeleton aggregates in the interior of the wound, rather than forming a ring that constricts around the wound, which highlights another of the many context-dependent differences observed with single cell wound healing (figure 1*d*) [28,29]. Once membrane resealing and cytoskeletal contraction around the wound have been accomplished the cell must remodel the affected membrane region and underlying cortical cytoskeleton to its pre-wounded state—a process that has remained largely elusive (figure 1*e*,*f*).

In the laboratory, researchers have generated cell wounds using sharp needles, nitrogen lasers or different kinds of drugs in tissue culture cells, yeast cells, *Xenopus* oocytes, sea urchin eggs, starfish eggs, *Dictyostelium* cells and *Drosophila* embryos [8,10–12,26,30–32]. These different systems yield highly similar results, yet offer unique and complementary features for studying cell wound repair, including the large size of *Xenopus* oocytes, the ease of *in vivo* imaging in *Xenopus* oocytes and *Drosophila* embryos, the genetic amenability of the *Drosophila* model and the translatability of human tissue culture cells.

In addition to being a physiological event of significant interest, single cell wound healing also represents a powerful, inducible system amenable to the study of complex signalling pathways and dynamic cytoskeletal rearrangements. It shares many features with other biological phenomena including cytokinesis and cortical flow, and may provide a new approach to the study of such processes, as well as a means to identify new genes/proteins involved in these processes. Cellular wound healing has been shown to be important during normal development, but it also underlies a broad range of pathologies. In certain cases, cells are unable to mount a substantial wound repair response in the face of regular wear-and-tear, which then contributes to the pathology of muscular dystrophies [33–35] and certain complications that arise from diabetes [33,36]. Conversely, some cell wound repair factors are upregulated in metastatic cancer cells, giving these cells an increased ability to migrate through dense extracellular matrix and invade new tissues [37–42]. In this review, we focus on the most recent findings in the field of single cell wound repair, with the goal of connecting these disparate developments to broader studies of different processes in basic science, as well as the pathology of certain human diseases.

## 2. How does a cell perceive that it has been wounded?

In their natural context, cells can be wounded by a multitude of different stresses, including mechanical and chemical assaults or by pathogens, resulting in wounds of different sizes and types. The cell, therefore, needs to be able to detect wounds of various origins and sizes and mount the appropriate response. A key challenge in determining the initiation cues for cell injuries is that the repair process is extremely rapid and that we are limited to defining the initiating events as the earliest step in the cell wound repair cascade that we are able to identify. Here, we review the evidence supporting the influx/interaction of the extracellular environment with the cells' interior as initiating events, and also examine other potential initiating events which have been proposed.

### 2.1. Initiating events of cell wound repair: calcium influx

**Figure 2.**
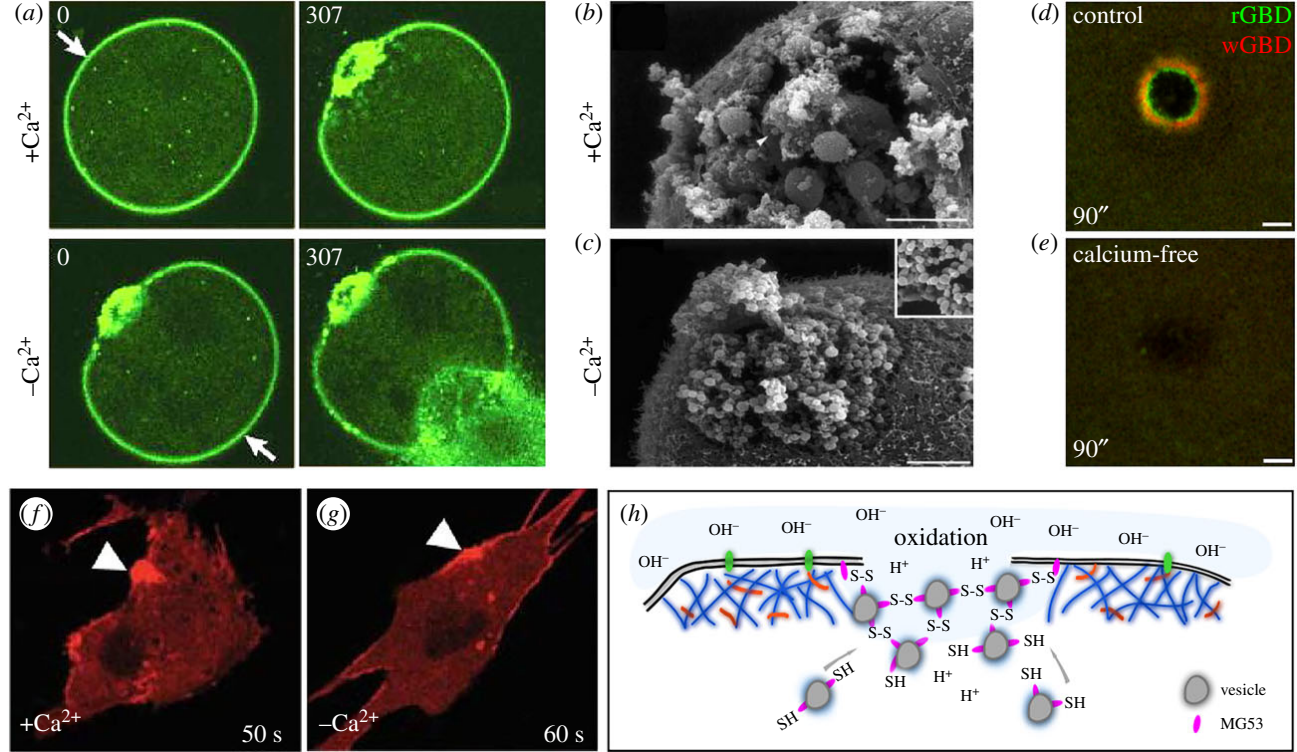
Calcium influx and oxidation are the most upstream events in cell wound repair. (*a*) Ca^2+^ dependence of cell wound repair. An FM1-43 labelled sea urchin egg was wounded twice with a laser. The first wound (arrow; upper left panel) was generated in the presence of calcium and exhibits a robust repair response (upper right panel). The sea urchin egg was moved to calcium-free sea water and a second wound (arrow; lower left panel) was then generated. In the absence of calcium no repair is initiated and cytoplasm can be seen flowing out of the wound (lower right panel). Adapted by permission from Springer Nature and Copyright Clearance Center: McNeil & Kirchhausen [4] (Copyright © 2005). (*b*,*c*) Scanning electron micrographs of sea urchin eggs in the presence (*b*) or absence (*c*) of calcium upon wounding. Wounds were generated mechanically using a needle. In the presence of calcium, vesicles (arrowhead) are recruited to the wound where they fuse to each other resulting in the formation of large vesicles. In the absence of calcium, no large vesicles are formed. Adapted by permission from Springer Nature and Copyright Clearance Center: McNeil & Baker [47] (Copyright © 2001). (*d*,*e*) Recruitment of activated Rho family GTPases in *Xenopus* oocytes upon wounding in the presence (*d*) or absence (*e*) of calcium. Activity biosensors for Rho (rGBD) and Cdc42 (wGBD) only exhibit distinct concentric ring patterns around the wound in the presence of calcium. Republished with permission of The Rockefeller University Press, from Benink & Bement; permission conveyed through Copyright Clearance Center, Inc. [24] (Copyright © 2005). (*f*,*g*) MG53 protein tethered to vesicles and membrane accumulates at wounds (arrowheads) even in the absence of calcium. Adapted by permission from Springer Nature and Copyright Clearance Center: Cai *et al.* [13] (Copyright © 2009). (*h*) Schematic depicting the role of oxidation in cell wound repair. Vesicles coated with MG53 protein are recruited to wounds where the MG53 proteins attach to each other through oxidation-dependent disulfide bond formation.

Cells, through the use of channels and pumps, maintain tight control of intracellular and extracellular ion concentrations [43–45]. When the plasma membrane is disrupted during wounding this strict control is lost: ions such as calcium, which are more concentrated in the extracellular environment, will immediately flow into cells despite the presence of cytosolic buffers that regulate calcium diffusion (figure 1*b*) [43–45]. The necessity of calcium influx for cell wound repair was first recognized in 1930 in the sea urchin model [46]. Calcium-dependent vesicle fusion at wounds was observed in sea urchin eggs and 3T3 cells in 1994 through the use of a calcium indicator and calcium media with various concentrations (figure 2*a*) [8]. Subsequently, similar experiments using a calcium indicator, calcium free media and/or calcium chelators (EGTA and/or BAPTA) have been performed in other cell types, including starfish eggs, *Xenopus* oocytes and tissue culture cells (figure 2*b–e*) [9–12]. The absence of calcium in those models causes severe delays and/or disruptions to wound repair as the influx of calcium induces downstream pathways needed to rapidly reseal the hole, as well as mediating membrane and cytoskeleton changes (see below). Through these studies and others, this calcium influx is the most conserved event observed at the onset of repair among species and in model systems, leading to its proposal as the initial repair trigger for cell wound repair.

The influx of calcium is thought to trigger wound repair by directly signalling through calcium responsive proteins. Recent studies in *Xenopus* and starfish have revealed that calcium influx also affects membrane potential state, elicits cytoskeleton changes and induces transcription [12,14,48–51]. In addition, membrane potential might be necessary for controlling ion levels through voltage-dependent channels to avoid cell death through excess calcium influx. Upon cellular wounding of *Xenopus* oocytes, electric current changes mediated by calcium influx occur in the outer cell membrane, generating a gradient of electrical current from the centre (−39.4 µA cm^−2^) to the outside of wounds (2.8 µA cm^−2^), whereas electrical current in normal, intact cell membrane is 0.7 µA cm^−2^. This current induces depolarization of the cell membrane that is gradually repolarized over several minutes. It is still unclear how these changes in membrane potential contribute to cell wound repair processes; however, membrane depolarization may be required for wound site recognition by transport vesicles and for the spatial localization patterns of proteins around wounds.

### 2.2. Initiating events of cell wound repair: oxidation

One of the systems in the body that most often undergoes cell repair is muscle cells, which are continuously exposed to large physical stresses. Unsurprisingly perhaps, recent studies have identified oxidation as a special trigger for wound repair in these cells through the study of the muscle-specific protein mitsugumin 53 (MG53). Upon cellular wounding, in addition to the influx of extracellular calcium, the largely reductive interior of the cell comes in contact with the oxidative extracellular environment [52]. MG53, a tripartite motif (TRIM) family protein (TRIM72) that is tethered to plasma membrane and intracellular vesicles as a monomer, oligomerizes by generating disulfide bonds between cysteine residues in response to oxidation exposure during cell wounding (figure 2*f–h*) [13]. Indeed, MG53 can accumulate at wounds in calcium free media, and MG53 mutant mice exhibit similar wound repair phenotypes regardless of calcium influx. Further, the inhibition of oxidation and extracellular calcium by DTT and EGTA in normal muscle fibre makes membrane repair worse in comparison with only the absence of calcium [13]. Thus, the oxidation that occurs from exposure to the extracellular environment not only leads to MG53 accumulation and oligomerization at wounds, but might be upstream or independent of calcium influx in these cells.

### 2.3. Initiating events of cell wound repair: other models

The cytoskeleton and its regulators control mechanical forces that change the shape and/or stiffness of cells in response to the extracellular environment. Mechanical forces are known to work as a trigger for many biological processes, including gene expression and protein transport [53–56]. For example, cellular stretching in lung epithelial cells induces exocytosis to increase membrane area, which is thought to be a protective response to this stress [32,57]. As the cell membrane is under tension [58,59], disruption of membrane and the underlying cortical cytoskeleton upon wounding will necessarily alter membrane tension at the wound. During wound repair, a decrease in membrane tension has been observed in 3T3 cells by tether force measurements (the distance that a membrane attached bead moves between unwounded and wounded states) [49]. In this case, exocytosis occurs calcium-dependently upon wounding and expands the membrane area by adding lipids to decrease the tension. Tension decrease is prevented in low calcium medium and this tension decrease is slower at a subsequent second wound, suggesting that membrane tension decrease is an active response to wounding. Indeed, induction of tension decrease in low calcium medium by cytochalasin D, which depolymerizes actin filaments, can improve membrane resealing [49]. While the tension decreases in this model support an already initiated wound repair process and are calcium-dependent, it is possible that the initial tension release inherent to making a hole in a membrane under tension may in itself initiate some events involved in cell wound repair. As tension sensing biosensors and the ability to assay tension in dynamic systems is refined, it will be interesting to revisit these questions.

Due to the many similarities with epithelial repair, transcriptional and translational responses to cell wound repair have also been considered. While transcription is known to play a significant role in epithelial repair [2,60], it is less likely to contribute as an initial signal for cell wound repair, as repairing wounds in single cells requires a very rapid response and is usually completed within tens of minutes [2,60]. Similarly, inhibition of protein translation in 3T3 cells did not affect initial wound repair events, however it did affect repair of a second wound: repair of the second wound was faster than the initial wound [50]. Thus, these functions are supportive: rather than initiating the process, they are required for providing potentiation to cells and/or supplying proteins that are consumed during wound repair processes.

## 3. Re-sealing the membrane breach

Continuity of the plasma membrane is essential to cell survival, acting as the primary barrier between the cytosol and extracellular space, and establishing electrical and chemical gradients required to drive normal cellular processes. Despite the different modes that initiate wound repair, it is clear that next major step towards cellular repair is the resealing of the plasma membrane (figure 1*c*). A number of responses for mending lesions in the membrane have been proposed, but no clear consensus of the mechanism(s) driving the repair process has been determined. Here we discuss the major hypotheses that have been postulated for how wounded cells reseal their membrane, and then highlight important protein-centric studies that are contributing significantly to our understanding of these processes.

### 3.1. Membrane ‘patch’ hypothesis

**Figure 3.**
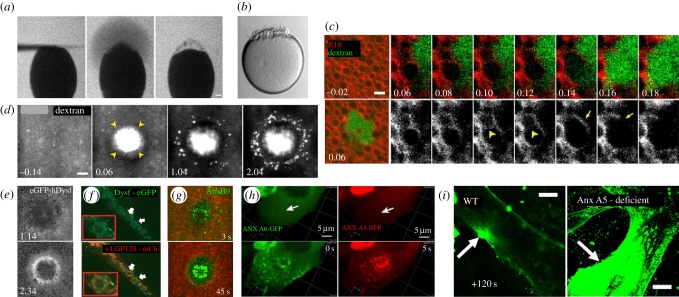
Mechanisms of plasma membrane (PM) repair. (*a*,*b*) Single cell repair in a sea urchin embryo. PM repair progression in a sea urchin egg after wound induction by a polylysine coated microneedle. (*a*) Sea urchin eggs were immersed in salt water containing 100 µg of fluorescein stachyose (FS). Lack of FS entry into the wounded egg indicates rapid plasma membrane resealing. (*b*) Sea urchin eggs establish a boundary at the wound site suggesting processive PM repair. Republished with permission of The Rockefeller University Press, from Terasaki *et al.*; permission conveyed through Copyright Clearance Center, Inc. [11] (Copyright © 1997). (*c*) Time-lapse micrographs of *Xenopus* oocytes wounded in the presence of dextran (green) after being stained with the PM marker, rhodamine B chloride (R18, red). Vesicle fusion and rupture along the wound edge establishes a single lipid bilayer (arrowheads at 10 s and 12 s show adjacent vesicle–vesicle fusion along the wound edge followed by rupture). (*d*) Wounding of *Xenopus* oocytes in the presence of dextran. Influx of dextran through the open central wound area (arrowheads) is observed by 6 s post-wounding. At later time points, dextran puncta are observed at the wound periphery of the initial wound opening indicative of exocytosis. (*e*) eGFP-human Dysferlin injected into *Xenopus* oocytes is recruited to a ring-like structure at the wound periphery and intracellular compartments upon wounding. Images (*c–e*) republished with permission of American Society of Cell Biology, from Davenport *et al.*; permission conveyed through Copyright Clearance Center, Inc. [20] (Copyright © 2016). (*f*) Dysferlin-eGFP (green) co-localizes with lysosomes (red) after mechanical wounding of L6 myotubes. Adapted from McDade & Michele, by permission of Oxford University Press [61] (Copyright © 2014). (*g*) Laser wounding of the *Drosophila* embryo shows annexin B9 (green) recruitment within the wound area. Image provided by M. Nakamura. (*h*) MCF cells showing annexin A6 and annexin A4 recruitment upon wounding. Arrows indicate the site of wounding (upper panels); annexin A6 showed an immediate recruitment followed by annexin A4 at 5 s. Adapted from fig. 6 in Boye *et al.*; Creative Commons license: https://creativecommons.org/licenses/by/4.0/ [62] (Copyright © 2017). (*i*) Myotubes were irradiated at the plasma membrane (arrow) in buffer containing 1 mM Ca^2+^ and 5 µg of FM1-43 dye. Annexin A5 deficient myotubes show an increase in FM1-43 uptake when compared to the control at 120 s. Adapted and reprinted from Carmeille *et al.*, with permission from Elsevier [63] (Copyright © 2016).

Early experiments carried out by McNeil and colleagues proposed a mechanism by which breaches of the membrane are rapidly re-sealed with a membranous patch [4,9,10,17]. Dubbed the ‘membrane patch hypothesis’, a mass of intracellular vesicles and/or membranous organelles are thought to accumulate at the wound site where they fuse to each other and the unwounded plasma membrane (figures 1*c* and 3*a*,*b*). It has been proposed that this membranous patch attaches to the plasma membrane first at discrete points around the periphery of the wound, followed by complete ‘vertex fusion’ (curvilinear zone of contact between the torn plasma membrane and the membranous patch along which the protein machinery needed for fusion becomes concentrated) to seal the wound. Early experiments supporting this hypothesis used sea urchin eggs and 3T3 cells loaded with lipophilic dye that showed exocytic vesicles were essential to plasma membrane repair [8]. Additionally, inhibition of SNARE activity via botulinum neurotoxin A indicated that fusion was necessary [8]. Recent work in the *Xenopus* oocyte also supports this model and went further to show that once the vesicle patch has attached to the unwounded plasma membrane, the vesicles lyse on the extracellular surface to form a single lipid bilayer, a phenomenon Bement and colleagues termed ‘explodosis’ (figure 3*c*) [20]. Thus, successive fusion of these intracellular compartments can reestablish membrane continuity, thereby preventing any further flux in or out of the cell. However, it remains unclear as to the exact source of the restorative membrane recruited to the wound in all contexts, as well as how conserved this source is among cells/species or in response to different types/sizes of wounds.

### 3.2. Exocytosis/endocytosis

An alternative hypothesis for membrane re-sealing has been proposed by Andrews and colleagues, involving the direct removal of the membrane lesion through exocytosis and subsequent endocytosis (figure 1*c*) [15,19,37,64–66]. A large number of exocytic vesicles were observed accumulating outside the wound site in cells when using markers specific for lysosomes, that were not previously observed due to the use of non-specific lipophilic dyes that labelled many intracellular organelles [67]. This observation led to the hypothesis that the plasma membrane at the wound site undergoes rapid and local exocytosis (and/or endocytosis) that excises the lesion such that the plasma membrane is re-sealed [67]. Consistent with this observation, wounding of sea urchin eggs and *Xenopus* oocytes has also been shown to induce rapid exocytosis of cortical granules (figure 3*d*) [9,10,20]. Further evidence suggests that the intracellular compartments undergoing exocytosis also serve a trafficking role to expel acid sphingomyelinase (ASM), a hydrolytic enzyme that converts sphingomyelin into ceramide at the external interface of the plasma membrane and induces endocytosis [68]. Ceramide lipid microdomains have been shown to contribute to membrane dynamics and promote inward budding, indicating a membrane priming step prior to endocytosis [69]. Loss of ASM showed no direct effect on exocytosis, but negatively affected the formation of endocytic vesicles and subsequent restoration of the plasma membrane. Rescue of ASM activity restored plasma membrane repair capabilities, further suggesting that endocytosis plays a role in plasma membrane repair [64,68]. Membrane resealing though endocytosis is limited by the ability of the endocytic compartment to remove the entirety of the lesion (maximum of 100 nm diameter [66]), thereby limiting it to smaller wounds. While a membrane patch composed of intracellular vesicles could theoretically function over a wider range of wound sizes, size constraints and/or other limitations of this process require further study.

### 3.3. Other plugging mechanisms

A number of alternatives have been proposed as mechanisms by which the cell can restore membrane continuity. One such mechanism focuses on the dynamics of the plasma membrane, suggesting that tension is the contributing factor of membrane resealing. A decrease in membrane tension was directly correlated with the restoration rate of the plasma membrane, suggesting that exocytosis was initiated to reduce tension and increase slack in the membrane to enable closure of plasma membrane disruptions [49]. Another alternative mechanism proposes that, rather than internalizing the disruption for degradation, membrane lesions are excised from the membrane by a process known as membrane blebbing [70]. Upon treatment with the streptolysin O (SLO) pore-forming toxin, it was observed by electron microscopy that SLO complexes became localized to membrane blebs protruding outward from the cell body. Ca^2+^ was not required for bleb formation, but was required later for its shedding into the extracellular space [70]. Other studies suggest that the lipid composition itself may play a role in recruiting downstream effectors needed to drive single cell wound repair. Lipid types were observed in distinct patterns at the wound site, and one lipid type in particular, diacylglycerol (DAG), was shown to be essential for sealing the plasma membrane [71].

### 3.4. Membrane plugging: insight from dysferlin and annexin studies

Dysferlin, a member of the ferlin protein family (single-pass transmembrane proteins with a short extracellular C-terminus and five-to-seven tandem C2 domains that differentially bind to Ca^2+^ on the cytoplasmic side), is recruited to cell wounds (figure 3*e*,*f*) [20,72,73]. Upon wounding, dysferlin deficient sarcolemma show an inability to properly drive plasma membrane repair [72] and have been suggested to play a role in promoting lysosomal exocytosis: dysferlin deficient cells show a significant reduction of the LAMP1 (lysosome) marker along the cell surface of injured myoblasts [74]. The C2 domains of dysferlin bind to the H3 SNARE domain of syntaxin 4, a ubiquitously expressed SNARE protein that facilitates lysosomal exocytosis, providing further evidence of the role of dysferlin in mediating lysosomal fusion during plasma membrane repair [61,75]. Dysferlin also interacts with MG53 via the dysferlin C2A domain [76]. MG53^–/−^ mutant mice show progressive muscle deterioration when exposed to more physical activity suggesting an inability to repair mechanical injury imposed upon the cell. Indeed, in muscle cells, dysferlin and MG53 form a shoulder structure at the wound site that is required for building up protein complexes needed for repairing wounds [29]. Although this shoulder structure has not yet been identified in other models, dysferlin might provide a scaffold function at wounds to facilitate protein recruitment and/or function.

Annexins (Anx) are a multifunction protein family that play an essential role in membrane repair and are characteristically defined by their ability to interact with negatively charged phospholipids in a Ca^2+^-dependent manner [63,77–80]. Anx B9, a *Drosophila* member of the annexin family, is rapidly recruited to the cell wound edge (within 3 s) where it is required for actin stabilization necessary for the recruitment of RhoGEF2 and subsequently Rho1 GTPase (figure 3*g*) [81]. Mammalian Anx A1, Anx A2 and Anx A6 are all recruited to the site of a wound upon laser ablation in a Ca^2+^-dependent manner to form a tight ‘repair cap’ (figure 3*h*) [29,82,83]. Anx A5 was also observed to rapidly respond to disruptions in the plasma membrane of human skeletal muscle cells, appearing at the wound edge within seconds independently of dysferlin (figure 3*i*) [63]. Experiments exploring the functions of Ca^2+^-binding domains and N-terminal domains responsible for oligomerization of Anx A4 provide evidence that the role of annexins in repair is not only Ca^2+^ dependent, but that they bind to the plasma membrane to induce an energetically favourable curvature relative to the size of the wound. Anx A6 also displayed similar properties of membrane dynamics, solidifying the role of annexins in plasma membrane repair [62]. Annexins have been suggested to serve as a linker between the plasma membrane and the underlying cytoskeleton via S100 proteins (Ca^2+^-EF binding domain containing proteins that exist as dimers), which can either function as regulators of calcium homeostasis within the cell or can be secreted to the extracellular space to serve alternative functions [84]. In particular, S100A11 co-localizes with Anx A2 upon wounding; depletion of either component fails to recruit the other.

## 4. Cytoskeletal responses in cell wound repair

A major step of cellular wound repair involves the coordinated regulation of the dynamic cortical cytoskeleton: the actin and myosin networks provide the driving forces for the repair processes (figure 1*d*). An enrichment of cortical F-actin to the wound periphery pervades repair responses in all observed animal models of single cell wound healing. In addition, regulators of actin polymerization, including profilin and Arp2/3, are enriched at the wound periphery and mediate F-actin polymerization [85,86]. Cortical flow, the translocation of the cortical cytoskeleton through localized movement towards the wound area, is observable in multiple systems. Cortical flow has been shown to be dependent on the Rho family GTPases in both the *Xenopus* and *Drosophila* models, and is hypothesized as the primary mechanism of actin accumulation at the wound edge [24,27,87].

**Figure 4.**
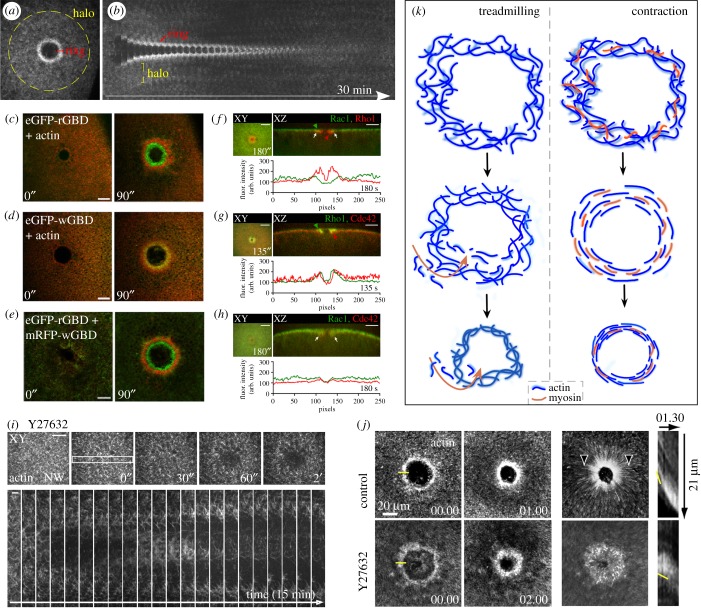
Cytoskeletal responses in cell wound repair. (*a*,*b*) Confocal XY projection at 180 s post-wounding (*a*) and kymograph (*b*) of NC4-staged *Drosophila* embryo expressing a GFP-actin reporter. Actin accumulates in two regions adjacent to the wound: a highly-enriched actin ring abutting the wound edge (red bracket), and an elevated actin halo encircling the actin ring (yellow circle). Membrane plug is also indicated. Adapted and republished with permission of The Rockefeller University Press, from Nakamura *et al.*; permission conveyed through Copyright Clearance Center, Inc. [81] (Copyright © 2017). (*c–e*) XY projections of the surface of a laser-wounded *Xenopus* oocyte displaying concentric rings of RhoA activity (green) (*c*), Cdc42 activity (green) alongside injected fluorescent actin (red) (*d*), and RhoA activity (red) overlaid with Cdc42 activity (green) (*e*). Republished with permission of The Rockefeller University Press, from Benink & Bement; permission conveyed through Copyright Clearance Center, Inc. [24] (Copyright © 2005). (*f–h*) XY and YZ projections alongside localized staining intensities across the wound midline of wounded *Drosophila* syncytial embryos for Rac1 (green) and Rho1 (red) (*f*), Rho1 (green) and Cdc42 (red) (*g*) and Rac1 (red) and Cdc42 (green) (*h*). Positions of GTPase recruitment (green or red arrowheads) and of GTPase co-localization (white arrows) are indicated. Adapted and reprinted from Abreu-Blanco *et al.*, with permission from Elsevier [27] (Copyright © 2014). (*i*) XY views and kymograph of laser-wounded *Drosophila* syncytial embryo expressing fluorescent actin reporters in the presence of Y27632. Adapted and republished with permission of The Rockefeller University Press, from Abreu-Blanco *et al.*; permission conveyed through Copyright Clearance Center, Inc. [26] (Copyright © 2011). (*j*) Time lapse images at 1 and 2 min after laser-wounded *Xenopus* oocytes following injection with control or Y27632 (Rok kinase inhibitor that prevents myosin-II activity) alongside a fluorescent actin reporter (left). Brightest points projection across experimental time representing cortical flow alongside a kymograph demonstrating wound closure (right). Yellow line at time 00:00 represents position of kymograph. Yellow line in kymograph identifies position of the leading edge. Adapted and reprinted from Burkel *et al*., with permission from Elsevier [25] (Copyright © 2012). (*k*) Schematic depicting XY view of actomyosin dynamics following wounding in the actin treadmilling and contraction wound closure models.

Within the *Xenopus* and *Drosophila* systems, recruited actin forms a discrete dense ring encircling the wound, with an ancillary halo of less dense actin (figure 4*a–d*) [12,26]. In other models, actin accumulates at the wound edge, but is less tightly organized [31,86]. Interestingly, within mammalian cell culture models, actin depolymerization is observed [49,88–90]. Local disassembly of cortical cytoskeleton is thought to facilitate access of trafficking repair machinery to the plasma membrane and to decrease adhesion between the membrane and cytoskeleton that may limit membrane healing. This may be achieved indirectly in larger wounds, as cortical cytoskeleton lost as a direct consequence of wounding is not immediately replenished, allowing membrane plug access to the cell surface. In embryonic models, proteases from the extracellular space may contribute to rapid deconstruction of wounded cytoskeleton, though this has not been tested [91].

In the *Drosophila* and *Xenopus* models, actin and myosin co-localize at the wound. While the actin and myosin co-translocate with wound closure in the *Drosophila* model, the actin and myosin arrays segregate as closure progresses in the *Xenopus* model, with myosin II localizing towards the interior of the wound [12]. Interestingly, wound closure in the *Dictyostelium* model does not require myosin: myosin II does not accumulate at the wound edge and repair dynamics are not altered in myosin II mutants. Within sea urchin coelomyocytes, actin accumulates at the wound edge alongside the Arp2/3 actin nucleating complex, and inhibition of actin polymerization disrupts wound healing, yet myosin II does not appear enriched at the wound edge [86]. Thus, despite the prevalence of actin accumulation at wounds in cell wound repair models, the means by which it functions in these different models appears to vary.

### 4.1. Rho family GTPase patterning primes assembly of wound repair players

Rho family GTPases are major cytoskeletal regulators, modulating actin–myosin filament formation via many mechanisms in a context-specific manner to control a wide spectrum of basic cell functions, including focal adhesion formation, lamellipodial extension, cell division and endocytosis [92–95]. Rho GTPases interact with a multitude of downstream effector proteins upon binding to GTP [95,96]. For example, Cdc42 acts upon WASP to promote actin nucleation via the Arp2/3 complex [97]. GTPase flux, continual switching between active and inactive states, is essential for expedient actin ring translocation and wound closure [98]. Rho family GTPase activity is promoted by RhoGEFs, which catalyse a switch from GDP to GTP, while GTPases are subsequently inactivated by RhoGAPs, which hydrolyse the GTPase-bound GTP to GDP [99,100]. Through constitutive activation or inactivation, these regulators contribute to the delineation of GTPase activity zones, and loss of MgcRacGAP has been shown to result in wider bands of Rho activity and disruptions to contractile array formation [98].

The Rho and Cdc42 GTPases were shown to form concentric arrays at the wound edge in the *Xenopus* oocyte model (figure 4*c–e*) [24]. Activated RhoA is found at the interior of the wound area and co-localizes with myosin II, whereas the external ring of Cdc42 colocalizes with actin [24]. These GTPases are negatively regulated by GAPs to maintain their exclusive zones. For example, Cdc42 is repressed in the wound interior by the GAP activity of Abr, which additionally promotes local RhoA activation through its GEF activity [101]. In *Drosophila*, Rho1 accumulates at the wound interior to the actin-rich ring, while Cdc42 has a broader overlap with actin towards the outside of the actomyosin ring, alongside diffuse bands of Rac1 and Rac2 (figure 4*f–h*) [27,81]. Loss of Rho function in *Drosophila* embryos results in increased Rac levels at the wound, suggesting crosstalk regulates GTPase patterning [27].

Rho family GTPase recruitment is fast: Rho1 is the first to accumulate to the wound at approximately 30 s, with Cdc42 and Rac1/2 zones appearing after approximately 90 s [27]. The molecular players bridging the 30 s gap between calcium influx and GTPase patterning remain an essential mechanistic question. A recent study showed this patterning is dependent on precursor RhoGEF localization to the appropriate GTPase zone: RhoGEF2, Pbl and RhoGEF3 pre-pattern the localization of the GTPases they regulate—Rho1, Cdc42 and Rac1, respectively [81]. The RhoGEFs require intact actin to form their discrete arrays, which is disrupted following actin depolymerization with latrunculin B, suggesting initial patterning is provided by Rho family GTPase-independent actin regulation [81]. Anx B9, a *Drosophila* member of the annexin family known to be involved in plasma membrane dynamics at the wound edge in other wounding models, is rapidly recruited to the wound edge in a pattern identical to that of Rho1 and RhoGEF2, which accumulates irrespective of an intact actin ring, and is required for correct RhoGEF2 localization, but not RhoGEF3 or Pbl. AnxB9 knockdown additionally disrupts actin accumulation at the wound edge, demarcating it as a precursor molecule for the RhoGEF/Rho GTPase patterning cascade [81].

Downstream of Rho1, effectors Rok and Dia exhibit identical staining patterns to Rho1 in accumulation at the wound interior, and are required for correct closure, potentially via their known roles in actin nucleation and contractile ring assembly, and the role of Rok in myosin II phosphorylation [27,102,103].

### 4.2. Divergent mechanisms in actin ring translocation: actomyosin ring contraction

Rho family GTPases through their downstream effectors facilitate phosphorylation of the myosin light chain to induce constriction of an actomyosin ring [104]. In the *Drosophila* model, the actomyosin ring encircling the damaged area contracts, causing uniform wound closure and reducing wound area (figures 1*d* and 4*k*) [22,26,27,105]. In this system, actomyosin ring translocation is inhibited by pharmacological inhibition of myosin II, indicating that the actomyosin ring is contractile in this model (figure 4*i*) [22,26]. Cortical cytoskeleton closure also draws the overlying plasma membrane closed. Connections between the contractile actomyosin ring and the overlying plasma membrane have been shown to be mediated, in part, by E-cadherin, in the *Drosophila* model [22,26]. It is tempting to ascribe mechanisms of actomyosin ring closure to that found in muscle sarcomeres or during cytokinesis where myosin II mediates sliding of antiparallel actin filament arrays [106]. While actomyosin rings during cytokinesis in yeast and mammalian cells do form antiparallel arrays [107], sarcomeric actomyosin levels remain constant, while cytokinetic rings reduce in size commensurate with closure, necessitating novel mechanisms of depolymerization which promote filament sliding without disrupting overall contractility through disrupting myosin motor function [108]. Yeast rings show a relative increase in myosin concentration as the ring circumference decreases [109]. In a Myo1 mutant lacking motor activity—where the rate of ring contraction is already decreased—the application of jasplakinolide further slowed or completely blocked ring closure, suggesting that myosin motor function contributes to, but is not required for, contraction. Taken together, actin depolymerization contributes to myosin-independent dynamics that is hypothesized to occur through continual shortening of individual contractile units [110,111]. Further studies are needed to determine the fate of the assembled actin and myosin arrays following wound closure.

### 4.3. Divergent mechanisms in actin ring translocation: actin treadmilling

Despite a presence of contractile myosin II within the actin ring that directs cortical flow [24,85], wounds in *Xenopus* oocytes are capable of closure following pharmacological myosin inactivation, demonstrating myosin independence, though this disrupts closure kinetics and organization of the actomyosin array (figures 1*d* and 4*j*,*k*) [25]. Observations of GTPase activity with fluorescent biosensors revealed that for both Cdc42 and RhoA, levels of activity were enriched at the leading edge of their zones, while being lost from the trailing edge [25]. The non-overlapping zones of GTPases alternately promoting interior actin polymerization and exterior disassembly facilitate the construction of consecutively smaller rings at the leading edge to decrease wound area, and ingress without myosin-mediated contraction. This actin treadmilling may ordinarily be masked or complemented by concurrent myosin-mediated contraction on the inner ring.

*Xenopus* oocytes are not the only model in which myosin-mediated constriction is dispensable for wound closure [25]. Injection of KT5926 into sea urchin coelomyocytes, ostensibly preventing myosin II phosphorylation and actomyosin contraction, failed to disrupt wound closure dynamics [86]. *Dictyostelium* wound healing also progresses as normal in *myo1* mutants [31]. Consistent with this, *Dictyostelium* cytokinesis, using a similar actomyosin ring as in other systems, was found to be myosin independent [112]. Evidence of actin treadmilling has not been shown in systems outside *Xenopus*, and it is likely from the differing actin organization that as yet unknown myosin-independent mechanisms of closure may be involved. Stemming from a simple organism, myosin-independent healing may represent an ancestral healing mechanism from which other models are derived, and which is usually masked in *Xenopus* by a predominant contraction. *Drosophila* cellularization, also dependent upon contractile actomyosin rings, goes through sequential myosin-dependent and -independent forms of contraction, which may be mechanistically similar to myosin-independent wound healing mechanisms in other systems [113].

### 4.4. Membrane and cortical cytoskeleton remodelling

After a cell's lesion has been sealed and drawn closed, it still has work to do to return to its pre-wounded state. These final steps of wound closure and cell regeneration are not well understood. Both the plasma membrane and the underlying cortical cytoskeleton need to be remodelled such that cells with very different functions and cell surface specializations can return to their former architectures and activities. Within the membrane patch hypothesis, the composition of lipids at the wound change first with the recruitment of the intracellularly-derived membranous plug and again as the patch is replaced with canonical cell plasma membrane. One hypothesis proposes that upon sufficient shrinkage of the wound area, the remaining lesion can be endocytosed using mechanisms similar to those defined for micropore closure [10,65]. While this may work well in smaller wounds, the recruitment and effectiveness of endocytic machinery or membrane-bending proteins is hypothetical in larger wounds, cholesterol-rich endocytic bodies have not been observed in the final stages of wound healing, and a combination of cortical closure and the membrane plug presumably would abrogate such mechanisms [68]. Mechanisms of cortical remodelling are also largely unknown. Advances in fluorescent reporters and *in vivo* imaging promise to make these processes more accessible to investigation.

## 5. Beyond cellular repair

**Figure 5.**
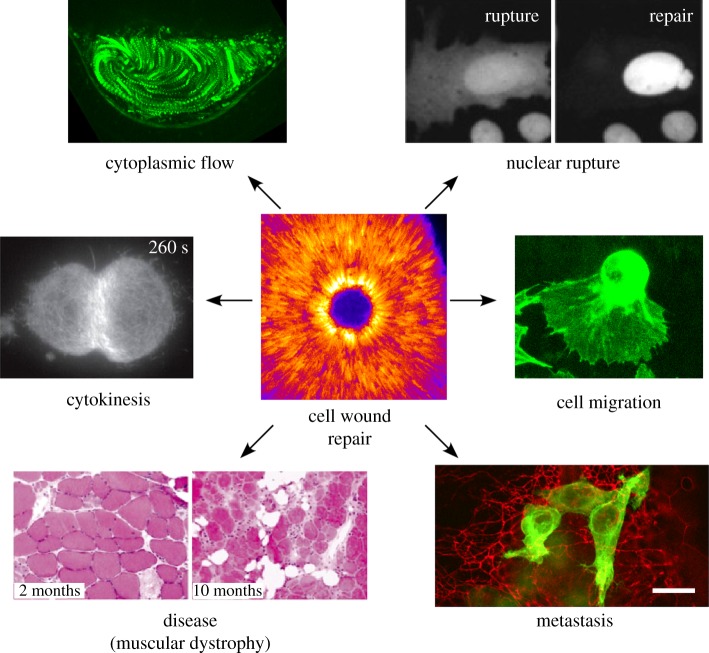
Beyond cellular repair. The study of single cell wound healing can inform many different fields of biology, from questions in basic science to the pathology of certain diseases. *Cell Wound Repair*: Single cell wound repair, shown here in a *Drosophila* embryo, requires the coordination of many cytoskeletal components, including spatio-temporal patterning of actin at the wound site. Image provided by M. Nakamura. *Cytokinesis*: Dividing cells require contraction of an actomyosin ring at the cell equator, shown here in human retinal cells undergoing cytokinesis. Adapted from fig. 4a in Spira *et al.*; Creative Commons license: https://creativecommons.org/licenses/by/4.0/ [114] (Copyright © 2017). *Cytoplasmic flows*: Cortical and cytoplasmic flows are responsible for bringing cellular components to sites where they are needed. During ooplasmic streaming in a developing *Drosophila* oocyte, cytoplasmic flows ensure that cellular content is evenly distributed. Image provided by J. Decker. *Nuclear Rupture*: Nuclei must faithfully repair after nuclear envelope rupture to preserve genomic integrity and this process may share protein components with cellular membrane repair. Nuclei from U2OS cells expressing GFP with a nuclear localization sequence undergoing nuclear rupture and repair. Adapted and republished with permission of The Rockefeller University Press, from Hatch & Hetzer; permission conveyed through Copyright Clearance Center, Inc. [115] (Copyright © 2016). *Cell Migration*: Cells, like this migratory haemocyte (macrophage) from a *Drosophila* larva, move throughout their environment by rapidly altering their cytoskeleton. Image provided by S. Parkhurst. *Metastasis*: Melanoma cells (green) spreading in a zebra fish larval hind-brain (red). In order for cells to invade new tissues, they must navigate a dense extracellular matrix while maintaining membrane integrity. Reprinted from Roh-Johnson *et al*., with permission from Elsevier [116] (Copyright © 2017). *Disease*: Skeletal muscle—a tissue that undergoes near constant wear and tear—from *Dysferlin* null mice, showing defective membrane repair in response to stress contributes to cell death. Adapted by permission from Springer Nature and Copyright Clearance Center: Bansal *et al*. [34] (Copyright © 2003).

Single cell wound healing also excels as an inducible model system for studying other subjects in basic science (figure 5). The clearest example of this is the study of the Rho GTPases. It has long been known that Rho GTPases are patterned in many cellular events (cell migration, cytokinesis, morphogenetic processes) [99,117], but often the study of these patterns is difficult in these systems. Single cell wound repair has many advantages for studying Rho GTPase patterning: (i) the pattern forms rapidly upon induction, (ii) the system itself is not moving (compared to a migrating cell), (iii) the patterning is largely in the view plane (versus cytokinesis), and (iv) similar protein cassettes are used in these processes. While it is undoubtedly true that differences between the process of single cell wound repair and these other cellular behaviours exist, single cell wound repair is an ideal model to interrogate big questions such as how Rho GTPase patterns can be formed, how these patterns can be refined into those with sharp boundaries, and how these patterns are maintained. Discovering answers to questions like these in single cell wound repair can provide new avenues of entry for studying Rho GTPase functions in other processes.

A related application is using cellular wound repair to study actin contractile dynamics. It has recently been shown that actomyosin contraction can occur differently depending on if the underlying actin structure is composed of disordered bundles, ordered bundles or disordered networks (cf. [106]). It is unclear how and/or when these differential architectures are used *in vivo*. Cellular wound repair is an ideal system to test hypotheses such as which form of actin structure underlies cell wound repair, and if genetic perturbations (via genetic screens) can alter the underlying actin structure and change contraction (thus healing) dynamics. Conclusions from studies on actin contractile dynamics would be widely applicable to many cell biological and developmental processes.

Another area of cellular wounding can inform the study of membrane dynamics, since it has recently been shown that the repair process needs both endocytosis and exocytosis pathways, cortical flow, and there is significant membrane remodelling that occurs in late stages of wound repair [15,19]. Indeed, wound repair is so intimately tied to membrane dynamics we suspect that any saturating screens for cell wound repair regulators will undoubtedly uncover new proteins that regulate membrane dynamics or at least new roles for known proteins. As such, single cell wound healing is a highly useful model for studying and discovering different pathways involving plasma membrane dynamics and the classes of molecules/machineries that affect these changes.

Cellular wound healing is a powerful system for studying a broad array of cellular processes, but also presents an opportunity to approach many human diseases from a new avenue since it has recently been appreciated that deficiencies in cellular wound repair, as well as an enhanced capacity to repair membrane lesions, contribute to many pathologies [3]. There are many diseases where compromised wound repair is implicated in the pathology, including diabetes, Niemann–Pick type A, Chediak–Higashi syndrome, and—most commonly—muscular dystrophies [33–36,118]. One tissue that regularly acquires damage at the single cell level is skeletal muscle, and it has been shown that mutations in certain muscle specific genes involved in calcium sensing and endocytosis, which are both required for cellular wound healing, lead to different muscular dystrophies [33–35]. Dysferlin, a calcium sensing sarcolemma protein, when mutated in the skeletal muscle, leads to human limb girdle muscular dystrophy (LGMD2B) [34,119]. Similarly, a mutation in muscle specific caveolin-3, a protein involved in endocytosis, leads to a different form of muscular dystrophy (LGMD1C) [120,121]. Skeletal muscle myopathy, which is a common complication arising from diabetes, has also been shown to occur because of a defect in cellular wound repair in this tissue [122,123].

Another possible entry point for studying wound repair and its relevance to disease is the role wound repair plays in cancer cell metastasis. As cancer cells invade tissues, they endure plasma membrane stress and damage as they navigate a dense extracellular matrix, and they must quickly heal membrane lesions to avoid excess calcium influx or leakage of cellular content, which both result in cell death [37,38,41,42]. It has been shown in human breast cancer cells that the S100A2–ANX A2 complex is upregulated, and is necessary for metastasis and invasion [124–126]. This protein complex responds to membrane lesions and promotes F-actin formation around the injury site, which is thought to provide a structural platform to anchor wound healing machinery and may also influence cytoskeletal activities that contribute to membrane resealing. In this case, restricting the cell's ability to undergo plasma membrane repair may be a potential therapeutic avenue, since the enhanced wound repair capacity of these cells appears to be bolstering their ability to invade new tissues.

## 6. Future perspectives

Robust wound repair mechanisms are vital in living organisms of all shapes and sizes to maintain the cell and tissue integrity needed to navigate the unrelenting physical and chemical assaults from their environments. A basic description of the events outlining single cell wound repair is in place from studies mainly in cultured cells, sea urchin eggs, *Xenopus* oocytes and *Drosophila* embryos. High power laser, needles and chemicals are used to generate wounds, but it is not yet known if these different types of wounds (e.g. burns versus rupture) elicit the same, partial, or completely different repair responses. Our knowledge of the signals and mechanisms governing single cell wound repair is still fairly limited, due in large part to the lack of information regarding the molecules/machineries involved in this process. And while we know a little about positive regulators for the repair process, we know even less about negative regulators—even though both of these would be potential targets for developing new strategies for treating cellular and/or tissue damage, for augmenting the effectiveness of existing treatment strategies, and for the advancement of regenerative medicine or tissue engineering where cell-based constructs are used to reconstruct tissues.

While tissues and organs in multicellular organisms consist of various types and numbers of cells that are attached to each other, all current models for the study of cell wound repair are using isolated cells, oocytes or embryos. In the context of wounds to a cell layer or tissue, damaged but repaired single cells may also function as important sentinels by playing key roles such as signalling to their neighbours to participate in the repair response. It will be important to take what is learned in these cell wound repair models to this greater landscape.

Although each section of this review has focused on the big picture physical mechanisms that underlie the cell's ability to repair itself, it will also be incredibly important to continue protein-centric studies. Largely, all evidence for how each of these aspects of wound repair are integrated into a cohesive response have come from studies interrogating the role of a single or few proteins. This includes the evidence that proteins like MG53 can be involved in both sensing the wound and helping to form the membrane plug or that proteins such as annexins may be one of the links between the membrane and cytoskeletal responses. It is essential that studies of both magnitudes continue in order to drive the cell repair field forward.

Here we have presented single cell wound healing as a highly applicable, inducible system that can be used to study a broad set of cellular processes in many different research organisms, and as a novel approach to the study of many human diseases. It will be interesting to see the new developments and mechanistic insights that arise in the field of single cell wound healing, and how these new findings may contribute to the investigation of basic biological processes, and how they may further our understanding and eventual treatment of human disease.

## Data accessibility

This article has no additional data.
